# Genome Sequence of an Epidemic Isolate of *Mycobacterium abscessus* subsp. *bolletii* from Rio de Janeiro, Brazil

**DOI:** 10.1128/genomeA.00617-13

**Published:** 2013-08-15

**Authors:** Rebecca M. Davidson, Paul R. Reynolds, Eveline Farias-Hesson, Rafael Silva Duarte, Mary Jackson, Michael Strong

**Affiliations:** Integrated Center for Genes, Environment, and Health, National Jewish Health, Denver, Colorado, USAa; Department of Pediatrics, National Jewish Health, Denver, Colorado, USAb; Instituto de Microbiologia, Universidade Federal do Rio de Janeiro, Rio de Janeiro, Brazilc; Mycobacteria Research Laboratories, Department of Microbiology, Immunology, and Pathology, Colorado State University, Fort Collins, Colorado, USAd; Computational Bioscience Program, University of Colorado Denver, School of Medicine, Aurora, Colorado, USAe

## Abstract

Multiple isolates of *Mycobacterium abscessus* subsp. *bolletii*, collectively called BRA100, were associated with outbreaks of postsurgical skin infections across various regions of Brazil from 2003 to 2009. We announce the draft genome sequence of a newly sequenced BRA100 strain, *M. abscessus* subsp. *bolletii* CRM-0020, isolated from a patient in Rio de Janeiro, Brazil.

## GENOME ANNOUNCEMENT

The nontuberculous mycobacterium (NTM) *Mycobacterium abscessus* is an environmental organism found in soil, water, and other ecological niches, and it acts as an opportunistic pathogen causing diseases ranging from cutaneous to pulmonary infections (1). Bacterial isolates of *M. abscessus* subsp. *bolletii* (formerly referred to as *M. abscessus* subsp. *massiliense*) (2) were linked to outbreaks of postsurgical skin infections in multiple Brazilian states from 2003 to 2009 (3–5). The epidemic isolates are collectively referred to as BRA100, as they exhibit a unique pulsed-field gel electrophoresis (PFGE) signature and show high levels of resistance to glutaraldehyde, the disinfectant used in most hospital sites that had reported cases (2, 4, 6).

The genome of one BRA100 strain, *M. abscessus* subsp. *bolletii* GO-06, from the Brazilian state of Goiás, was previously released (7), but the sequencing of additional outbreak strains is needed for comparative studies of isolates from different geographic regions. Here, we present the draft genome sequence of a BRA100 strain from Rio de Janeiro, Brazil, called *M. abscessus* subsp. *bolletii* CRM-0020, which was isolated in 2006 from a soft tissue biopsy specimen (4). The PFGE profile of CRM-0020 showed 13 bands by DraI digestion similar to those of the outbreak strains isolated in the Brazilian states of Goiás and Pará (4).

The genomic sequence data of CRM-0020 were obtained with Illumina MiSeq (150 bp, paired-end) at 130× coverage and with Roche 454 GS-FLX Titanium (average length, 604 bp) at 10× coverage. MiSeq reads were assembled into contigs with the software packages A5 (8) and Velvet (9) that were trimmed into 400-bp pseudo-Sanger reads. A combination of pseudo-Sanger, 454, and MiSeq reads were combined for hybrid assembly using Newbler (Roche). The contigs were ordered by alignment to the *M. abscessus* subsp. *abscessus* reference genome ATCC 19977 (10) with Mauve 2.3.1 (11), and genomic features were predicted and annotated using the NCBI Prokaryotic Genome Automatic Annotation Pipeline.

The draft genome of CRM-0020 consists of 44 contigs with an average contig length of 111,245 bp, a total size of 4,840,000 bp, and a G+C content of 64.3%. A total of 4,750 coding sequences (CDSs) were predicted, including 3,378 CDSs (71.1%) with functional annotations and 1,372 CDSs (28.9%) that were annotated as hypothetical proteins. The genome contains 46 tRNAs and one rRNA cistron. A comparative analysis between CRM-0020 and ATCC 19977 revealed that 4,189 CDSs (88.2%) are shared between the two strains, while 561 (11.8%) CDSs are unique to CRM-0020. A single contig (length, 56,466 bp) aligned with 99% identity to the *M. abscessus* subsp. *bolletii* plasmids, pMAB01 (12) and BRA100 (GenBank accession no. CP003505), and includes 63 predicted CDSs.

Whole-genome sequence alignments revealed 6,217 single nucleotide polymorphisms (SNPs) (1.28 SNPs per Kb) between CRM-0020 and GO-06, showing a low level of divergence among the regional outbreak strains from Brazil. In contrast, 49,354 SNPs (10.2 SNPs per Kb) were observed between CRM-0020 and the type strain of *M. abscessus* subsp. *bolletii* CCUG 48898 (13), revealing a higher level of divergence between unrelated strains than between outbreak strains.

### Nucleotide sequence accession numbers.

This whole-genome shotgun project has been deposited at DDBJ/EMBL/GenBank under the accession no. ATFQ00000000. The version described in this paper is version ATFQ01000000.
